# Development of a Specialized Telemedicine Protocol for Cognitive Disorders: The TeleCogNition Project in Greece

**DOI:** 10.3390/geriatrics10040094

**Published:** 2025-07-16

**Authors:** Efthalia Angelopoulou, Ioannis Stamelos, Evangelia Smaragdaki, Kalliopi Vourou, Evangelia Stanitsa, Dionysia Kontaxopoulou, Christos Koros, John Papatriantafyllou, Vasiliki Zilidou, Evangelia Romanopoulou, Efstratia-Maria Georgopoulou, Paraskevi Sakka, Haralampos Karanikas, Leonidas Stefanis, Panagiotis Bamidis, Sokratis Papageorgiou

**Affiliations:** 1 1st Neurology Department, Aiginition University Hospital, National and Kapodistrian University of Athens, 11528 Athens, Greece; angelthal@med.uoa.gr (E.A.); j-stam@hotmail.gr (I.S.); eua_smaragdaki@hotmail.com (E.S.); kalliopivourou@gmail.com (K.V.); eva.st.92@gmail.com (E.S.); d.kontaxopoulou@hotmail.com (D.K.); chkoros@gmail.com (C.K.); jpapatriantafyllou@gmail.com (J.P.); lstefanis@med.uoa.gr (L.S.); 2 Laboratory of Medical Physics and Digital Innovation, School of Medicine, Aristotle University of Thessaloniki, 54636 Thessaloniki, Greece; vickyzilidou@gmail.com (V.Z.); evangeliaromanopoulou@gmail.com (E.R.); pdbamidis@gmail.com (P.B.); 3 General Hospital of Leros, 85400 Lakki, Greece; evageorgpap@gmail.com; 4 Athens Association of Alzheimer’s Disease and Related Disorders, 11636 Marousi, Greece; info@psakka.gr; 5 Department of Computer Science and Biomedical Informatics, University of Thessaly, 35131 Lamia, Greece; karanikas@uth.gr

**Keywords:** telemedicine, telemedicine protocol, cognitive disorders, dementia, telecare, remote, specialized care

## Abstract

Background/Objectives: Access to specialized care for patients with cognitive impairment in remote areas is often limited. Despite the increasing adoption of telemedicine, standardized guidelines have not yet been specified. This study aimed to develop a comprehensive protocol for the specialized neurological, neuropsychological, and neuropsychiatric assessment of patients with cognitive disorders in remote areas through telemedicine. Methods: We analyzed data from (i) a comprehensive literature review of the existing recommendations, reliability studies, and telemedicine models for cognitive disorders, (ii) insights from a three-year experience of a specialized telemedicine outpatient clinic for cognitive movement disorders in Greece, and (iii) suggestions coming from dementia specialists experienced in telemedicine (neurologists, neuropsychologists, psychiatrists) who took part in three focus groups. A critical synthesis of the findings was performed in the end. Results: The final protocol included: technical and organizational requirements (e.g., a high-resolution screen and a camera with zoom, room dimensions adequate for gait assessment, a noise-canceling microphone); medical history; neurological, neuropsychiatric, and neuropsychological assessment adapted to videoconferencing; ethical–legal aspects (e.g., data security, privacy, informed consent); clinician–patient interaction (e.g., empathy, eye contact); diagnostic work-up; linkage to other services (e.g., tele-psychoeducation, caregiver support); and instructions for treatment and follow-up. Conclusions: This protocol is expected to serve as an example of good clinical practice and a source for official telemedicine guidelines for cognitive disorders. Ultimate outcomes include the potential enhanced access to specialized care, minimized financial and logistical costs, and the provision of a standardized, effective model for the remote diagnosis, treatment, and follow-up. This model could be applied not only in Greece, but also in other countries with similar healthcare systems and populations living in remote, difficult-to-access areas.

## 1. Introduction

The prevalence of dementia is rising, especially among aging populations, posing an important challenge to health systems worldwide [1]. Despite the substantial scientific developments in diagnosis and treatment approaches, patients with cognitive disorders in remote and underserved areas often face significant difficulties in accessing appropriate care [2]. Geographical barriers, mobility problems, limited resources, and the scarcity of specialized neurologists, neuropsychologists, and other healthcare professionals in rural areas can impede prevention, accurate and timely diagnosis, treatment, and monitoring [2]. Therefore, there is an urgent need for innovative solutions to address this gap and ensure equitable accessibility to specialized services [3].

Telemedicine, as defined by the World Health Organization (WHO), refers to the delivery of healthcare services using information and communication technologies (ICT) for diagnosis, prevention, and treatment, where distance is a critical factor [4]. Telemedicine falls under the umbrella of “telehealth,” which encompasses not only remote clinical care but also medical education and research as its three key pillars. There are several forms of telemedicine services, including synchronous (real-time), asynchronous (store-and-forward), and remote monitoring applications [4].

Real-time videoconferencing supporting live interaction between clinicians and patients has shown promising potential in cognitive disorders [5]. Dementia diagnosis relies heavily on medical history, which can be obtained remotely, making telemedicine a valuable alternative [5]. Most parts of the neurological and neuropsychological examination can be performed through telemedicine with appropriate adaptations, except for muscle tone, reflexes, and postural instability in the absence of a local trained healthcare professional (LTHP) [6]. Alzheimer’s disease (AD), dementia, and, to a lesser extent, mild cognitive impairment (MCI) can be diagnosed via telemedicine, with diagnostic accuracy comparable to in-person evaluations [7]. Access to specialized care allows for an accurate diagnosis, especially for atypical or early-onset forms of dementia, which require evaluation from specialized professionals [8]. Telemedicine use can result in reduced transportation distance, time, and costs, fewer visits to emergency departments and preventable hospitalizations, protection from infectious diseases, improved patients’ follow-up and treatment adherence, as well as decreased caregiver stress [9]. Importantly, several studies have demonstrated high acceptability and satisfaction with remote examinations among patients, caregivers, and healthcare providers, with convenience, better access to specialized care, and lower cost mentioned as some of the most important benefits [9,10].

High-quality healthcare is essential for ensuring optimal patient care, improving the quality of life, and promoting the efficiency of healthcare systems [11]. According to the WHO, quality of healthcare is defined by seven key dimensions: effectiveness, safety, people-centeredness, timeliness, equitability, efficiency, and integrated care [11]. These aspects serve as a foundation for delivering comprehensive and patient-centered medical services, as well as addressing disparities in access to care [11]. Based on our recent review, telemedicine can enhance all these aspects [9]. However, despite the growing use of telemedicine, a standardized and comprehensive protocol that aligns with these seven dimensions for dementia is lacking. Current telemedicine approaches often vary in structure, assessment methods, and clinical rigor, potentially leading to inconsistencies in diagnosis and patient management [7]. In addition, apart from the recommendations for the remote neurological examination suggested by the American Academy of Neurology (AAN) and other organizations [5,12,13], no standardized protocol or official guidelines focusing on cognitive disorders have been developed yet.

Greece has over 100 inhabited islands, primarily in the Aegean Sea, with distances from Piraeus ranging from 50 to 550 km [14]. Many health centers are understaffed, forcing patients to travel long distances to reach specialized care, while weather-related travel restrictions often cause additional delays [15]. Most islands lack dementia specialists, relying instead on general practitioners and rural doctors in local health centers [14,15]. The National Telemedicine Network (NTN), established in 2016 by the 2nd Regional Healthcare Administration of Piraeus and Aegean, constitutes the most comprehensive telemedicine initiative in Greece. It consists of approximately 60 telemedicine stations in the Aegean islands’ health centers and regional hospitals, facilitating remote consultations across multiple specialties, thereby improving access to specialized care [14].

In this context, the “Specialized Outpatient Clinic of Memory, Dementia, and Parkinson’s Disease through the NTN” (DEM-NTN) was established at the Neurology Department of the Aiginition University Hospital in Athens in March 2021 in collaboration with the 2nd Regional Healthcare Administration of Piraeus and Aegean [14]. This tertiary clinic provides consultations via videoconferencing for patients with cognitive and/or movement disorders at health centers of underserved Aegean islands by neurologists, neuropsychologists, and psychiatrists specializing in these diseases. In addition, it contributes to the education of local healthcare professionals in the neurological and neuropsychological examination and management of patients with cognitive and movement disorders. By December 2024, more than 300 telemedicine visits had been conducted, with high levels of satisfaction among patients, caregivers, and healthcare professionals, highlighting the feasibility and acceptance of telemedicine within the Greek healthcare landscape. Additional telemedicine services in Greece for dementia and/or psychogeriatric conditions include those from the Athens Alzheimer’s Association and the INTegRated InterveNtion of pSychogerIatric Care (INTRINSIC) program [15]. However, DEM-NTN is the only tertiary clinic within the Greek national telemedicine network dedicated to patients with cognitive or movement disorders, at which, for the past four years, specialized neurologists, psychiatrists, and neuropsychologists have been providing regular, comprehensive remote assessment and management.

Building upon these gaps and opportunities, research project “Development and Evaluation of an Innovative Telemedicine Model for Patients with Cognitive Disorders in Remote Areas—TeleCogNition,” funded by the Hellenic Foundation for Research and Innovation (HFRI) was initiated in 2023, aiming to create and evaluate a specialized telemedicine assessment protocol for patients with cognitive disorders residing in remote regions of Greece. The objective of the present study was to develop a comprehensive, specialized telemedicine protocol for the neurological, neuropsychological, and neuropsychiatric assessment of patients with cognitive disorders. This model, constituting a part of the “TeleCogNition” project, focuses on real-time telemedicine provided by specialists to patients in remote healthcare facilities via videoconferencing.

While the NTN and affiliated initiatives, including the DEM-NTN, have provided valuable services, including our team’s own ongoing contributions, “TeleCogNition” was developed to formalize, structure, and evaluate a comprehensive protocol that can serve as a standardized model, addressing the current lack of national and international official guidelines for the tele-assessment of cognitive disorders. While INTRINSIC and other psychogeriatric or memory services such as those of the Athens Alzheimer’s Association provide psychosocial support and basic telemedicine services, TeleCogNition innovates by (i) developing a multidimensional clinical protocol that includes neurological, neuropsychological, and neuropsychiatric components adapted for telemedicine use, (ii) incorporating specific organizational, ethical–legal, technical, and clinician–patient communication components aligned with the WHO’s seven quality-of-care dimensions, (iii) utilizing a three-layered methodology (literature review, real-world experience from the DEM-NTN, and structured input from national focus groups) to build an evidence-based and field-tested model, and (iv) being the first structured effort to pilot and evaluate such a comprehensive protocol for cognitive disorders in remote Greek populations.

## 2. Materials and Methods

### 2.1. Overview

**Figure 1:**
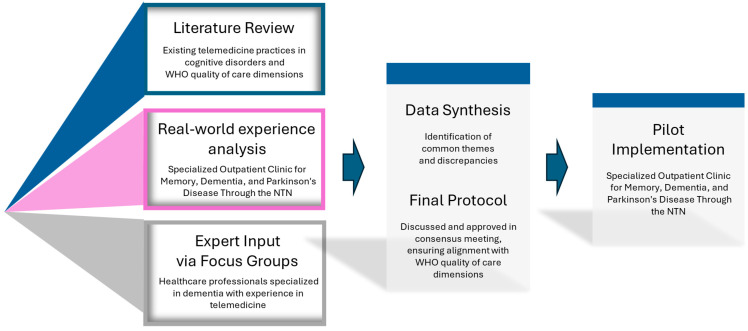
Schematic overview of the methodological approach for the development of a telemedicine protocol for cognitive disorders.

Our methodology combined three key elements: (i) a comprehensive literature review, (ii) our practical three-year experience gained from the DEM-NTN, and (iii) three (3) focus groups with healthcare experts in dementia and experience in telemedicine. The final protocol was developed based on the critical synthesis of the data obtained from these three (3) sources (Figure 1).

### 2.2. Literature Review

A literature review was conducted in MEDLINE (PubMed), Scopus, and Google Scholar to identify evidence-based practices, the existing guidelines, and relevant research on telemedicine for cognitive disorders. Peer-reviewed articles written in Greek or English without any time restrictions were searched independently by our team, consisting of neurologists and neuropsychologists with experience in cognitive disorders and telemedicine (E.A., E.S., D.K., E.S., I.S., K.V.). The keywords included such terms as “telemedicine,” “telecare,” “teleconsultation,” “remote,” “digital,” “videoconferencing,” “cognitive disorders,” “cognitive impairment,” “dementia,” “neurological examination,” “neuropsychological,” “neuropsychiatric,” “tele-psychoeducation,” “healthcare quality,” and “WHO,” in various combinations. The focus was on studies investigating the feasibility and reliability of remote neurological, psychiatric, and neuropsychological assessment, the development of telemedicine services for cognitive disorders (including organizational, legal, ethical, and technological aspects), quality of healthcare according to the WHO, as well as non-pharmacological interventions such as tele-psychoeducation and remote support programs for caregivers. Titles and abstracts were screened, followed by a full-text review of selected articles. A snowballing technique was applied to identify additional articles from the bibliographies of relevant studies. Furthermore, we communicated with stakeholders in public health, digital health, and dementia to gather additional relevant information.

Data synthesis was performed by our team, and the findings were integrated based on the main sub-areas of the telemedicine visit.

### 2.3. Experience from the “Specialized Outpatient Clinic for Memory, Dementia, and Parkinson’s Disease Through the National Telemedicine Network”

Our team (S.P., E.A., D.K., E.S., E.S., K.V., I.S.) leveraged the three-year clinical experience gained from the ongoing operation of the DEM-NTN at the Aiginition University Hospital in Athens (March 2021—March 2023) [14]. Referrals were typically initiated by general practitioners who identified patients with suspected cognitive impairment or movement disorders.

Prior to each telemedicine session, patients underwent a preliminary assessment at their local health center, including the collection of basic demographic information and short medical history, list of medications, and any potential useful information such as prior neurological evaluations. These data, along with any relevant medical records, were sent to the physicians of the outpatient clinic via the NTN.

A telemedicine session, which lasts approximately one hour, includes a detailed evaluation, including medical history from the patient and the informant (focusing on cognitive and behavioral symptoms and functionality), neurological examination adapted for remote settings, as well as neuropsychological and neuropsychiatric assessment (using validated instruments that could be administered via videoconferencing). The videoconferencing equipment of the NTN was used, consisting of a high-resolution camera and a screen.

During the operational period of the DEM-NTN, specific procedures were selected and adapted for the remote evaluation. Various technical challenges related to videoconferencing equipment and data transmission were also identified and addressed. Important lessons were learned regarding communication with patients and caregivers for enhancing their engagement. Based on these observations, the workflow was optimized for the referral process, virtual examination, data transmission, reporting of results, and collaboration with local healthcare professionals and other services. Connection via national network “Syzefxis” and the implementation of privacy protection measures optimized data security.

By December 2024, a total of 326 telemedicine visits (172 first and 154 follow-up assessments) had been conducted, with high acceptance among users. Patients, caregivers, and healthcare professionals were highly satisfied with this service in terms of convenience, improved access to specialized care, reduced cost, clinician–patient communication, and better health, as described in our previous study [14].

A structured approach was applied for the documentation of the experience from the DEM-NTN based on the different sub-areas of the telemedicine visit. A consensus meeting was held among the members of our team to discuss individual observations during telemedicine visits, synthesize and summarize the collective findings.

### 2.4. Focus Groups

To gather expert perspectives and ensure the practical applicability of the protocol, three focus groups were conducted with healthcare professionals specializing in dementia care and having practical experience in telemedicine. Participants were carefully selected to represent a diverse range of disciplines (neurologists, psychiatrists, and neuropsychologists) and various geographical regions across Greece.

First, a structured questionnaire consisting of fifteen closed-ended, multiple-choice questions was developed to gather preliminary insights from the participants’ baseline perceptions and priorities, focusing on the neurological, neuropsychological, and neuropsychiatric examination, referral plans, and diagnostic certainty. The questionnaire was filled out anonymously and served as a preparatory tool for the focus groups, enabling the identification of areas of consensus and divergence before the in-depth discussions. Descriptive statistics were used for the presentation of the questionnaire’s results, including frequencies for categorical variables and means and standard deviations for quantitative variables.

Each focus group was structured to facilitate open dialogue, exchange of knowledge and experience, as well as discussion of best practices to identify the key considerations for the protocol. A semi-structured interview guide consisting of 12 questions belonging to five (5) telemedicine sub-areas was created, based on the literature review and preliminary discussions within our team (Table 1).

The focus group sessions were guided by trained moderators (E.A., D.K.) who facilitated the discussion, maintained a respectful environment, and ensured that all the participants had an opportunity to participate. Two experienced note-takers (E.R., V.Z.) documented notes on the main themes, recurring issues, and recommendations. Each session lasted approximately 90–120 min, carried out online via the Zoom platform. The participants were provided with clear instructions on the purpose and their role in the TeleCogNition project. In addition, the seven (7) dimensions of healthcare quality were described at the beginning of the sessions, emphasizing that the discussion and suggestions should agree with this framework. Informed consent was obtained from all the participants prior to their participation and video recording, and anonymity and confidentiality were assured.

Two experienced researchers (V.Z., E.R.) analyzed the qualitative data from the focus group sessions using thematic analysis, by identifying key and recurring themes and patterns and developing a coding scheme. The identified themes were synthesized according to the structure of the different telemedicine sub-areas.

### 2.5. Data Synthesis

For each telemedicine sub-area (e.g., technical aspects, neurological examination, neuropsychological testing), results from the literature review, experience from the DEM-NTN, and focus groups were critically synthesized and integrated. Our team (S.P., E.A., D.K., E.S., E.S., K.V., I.S.) identified common themes and discrepancies among these three sources. A structured consensus meeting was held between the members of the research team of the TeleCogNition project to discuss and resolve the areas of disagreement and develop the final protocol, ensuring also that all the included elements aligned with the WHO’s seven (7) dimensions of healthcare quality.

### 2.6. Pilot Implementation Phase in the “Specialized Outpatient Clinic for Memory, Dementia, and Parkinson’s Disease Through the NTN”

The new protocol was implemented in the DEM-NTN from 01.09.2024 to 30.09.2024 to identify its feasibility, acceptability, and areas for improvement. During this pilot phase, 12 telemedicine visits were conducted. Each visit adhered to the finalized protocol, and observations for the evaluation of its performance were recorded by our team, based on the predefined telemedicine sub-areas.

## 3. Results

The results from the literature review and the DEM-NTN experience have already been synthesized and presented in the form of a proposed guide elsewhere [16]. Building on this foundation, we present herein: (i) the results from the preliminary questionnaires filled out by the focus group participants, (ii) the synthesized findings for each telemedicine sub-area based on a three-layered approach (literature review, DEM-NTN experience, focus groups), focusing on areas of agreement, discrepancies, and emerging issues, (iii) the structure of the final protocol, and (iv) the results from its pilot implementation in the DEM-NTN.

### 3.1. Focus Group Participants’ Characteristics

A total of 15 healthcare professionals (9 women, 6 men) with expertise in dementia and experience in telemedicine participated in the focus groups. The first group included 5 members (2 psychiatrists, 2 neurologists, and 1 neuropsychologist), the second had 4 members (2 psychologists, 1 neuropsychologist, and 1 neurologist), and the third comprised 6 members (1 neurologist and 5 neuropsychologists). The participants were affiliated with either General or University Hospitals (53.3%) or dementia daycare centers (46.7%) and were based in Athens, Thessaloniki, Alexandroupolis, Ioannina, Larissa, and Iraklion. The average years of experience in cognitive disorders/dementia and telemedicine were 15.55 ± 9.15 and 2.82 ± 2.14 years, respectively.

### 3.2. Responses to the Preliminary Questionnaires

The responses of the focus group participants to the preliminary questionnaires are presented in Table 2. All the participants agreed on the need for a specialized telemedicine protocol for cognitive disorders (100%). Regarding informed consent, 75% of the physicians obtained verbal consent and 25% written, while 85.7% of the psychologists/neuropsychologists required written consent. The physicians unanimously required caregiver presence during evaluations (100%), while the psychologists/neuropsychologists varied (28.6% required, 28.6% did not, and 42.9% assessed on a case-by-case basis). Mobility assessments were conducted systematically or occasionally by the physicians (50% each). Mini–mental state examination (MMSE) was the most used screening tool by both groups, with functional and neuropsychiatric scales also widely applied. Satisfaction questionnaires were routinely distributed by the physicians (100%) but rarely by the psychologists/neuropsychologists (14.3%). Diagnostic accuracy was perceived as minimally affected (<20%) by the remote settings in most cases.

### 3.3. The Final Form of the Comprehensive Telemedicine Protocol for the Assessment of Patients with Cognitive Disorders

The synthesized findings for each telemedicine sub-area are described, according to the literature review, the DEM-NTN experience, and the quantitative and qualitative data from focus groups.

#### 3.3.1. Organizational Aspects

The literature review, focus groups, and DEM-NTN experience highlighted the importance of adequate room dimensions enabling gait assessment for at least five steps in front of the camera view [16]. One participant mentioned that “a quiet and private environment is essential for ensuring a good quality of communication.” A well-lit space, good ventilation, essential items available such as a pen and paper, and printed forms of neuropsychological testing were also emphasized. One participant noted that “no visible clocks or calendars should be close to the patient during the neuropsychological examination,” with “necessary personal aids, such as glasses and hearing devices, being directly available.” These comments agree with both the literature and our experience [5,16]. Additional elements of the final protocol that were not mentioned in the focus groups but were identified in the literature included a comfortable chair for the patient, the router being close to the examination room, and all the participants being fully visible in the camera field of view [16]. According to our experience, a digital blood pressure monitor should be available in the room for assessing pulse, blood pressure, and orthostatic hypotension when needed. Our team determined that a pre-filled document with a patient’s medical data should be sent to the remote specialists. The maximum duration of each visit is proposed to be 60 min, while additional neuropsychological assessment could be arranged on a second visit, when needed. A remote specialized team should include at least one neurologist, one psychiatrist, and one neuropsychologist with experience in cognitive disorders [16]. If evaluation or intervention by another professional (e.g., speech therapist) is needed, a referral should be made for an in-person or telemedicine consultation.

#### 3.3.2. Technical Aspects

Literature review, focus groups, and DEM-NTN experience agreed that technology-related factors are very important for the feasibility, reliability, and acceptability of telemedicine visits [5,12,13,16]. These factors included the stability of the Internet connection to avoid distractions, the availability of suitable equipment with a proper audio–visual set-up with good acoustics and resolution at both the patient’s and the remote expert’s locations, user-friendly interfaces, and technical support for addressing technical difficulties [5,12,13,16]. Both the literature and the DEM-NTN experience also highlighted the need for optical camera zoom with rotating ability, stable connectivity (especially for assessing mild bradykinesia and tremor—due to their rhythmic nature), noise-canceling microphones, data sharing with sufficient bandwidth between local and remote providers, ideally screensharing for the neuropsychological assessment, and also two screens for simultaneous examination and data documentation to maintain eye contact with the patient at all times [12,16].

#### 3.3.3. Initiation of a Telemedicine Session

All three information sources generally agreed on the initial words and steps for the telemedicine session (see below for ethical and legal issues). Before the initiation of a telemedicine session, written informed consent should be obtained from the patient or the legal representative. According to the literature and the DEM-NTN, at the beginning of a session, the remote specialist greets everyone, ensures they can hear properly, and welcomes the patient and other participants [12,16]. They introduce themselves, stating their name, role, and clinic, and introduce the rest of the team. The patient’s name and date of birth are verified, and the names of other attendees are recorded. The patient’s address and phone number are noted for emergency cases. The physician asks about any vision or hearing impairments and advises using glasses or hearing aids if needed. If a healthcare professional is present with the patient, the physician requests their assistance during the session or gathers relevant medical information before they leave. The patient is asked to have an A4 sheet and a pen ready for any writing or drawing tasks [12,16]. Finally, the patient is asked for consent for their family member/caregiver to remain during the session.

#### 3.3.4. Medical History

While the literature supports the feasibility of the remote neurological history [5,12], our focus groups highlighted some key aspects, which were also identified by our team. Obtaining medical history from both the patient and the caregiver is a necessity in cognitive disorders, as also mentioned during the focus groups. The medical and family history should be obtained as it would be in an in-person visit. However, telemedicine creates significant challenges. First, it is emphasized that patient permission is always required before speaking privately with caregivers. In an in-person visit, the patient can undergo neuropsychological testing in a separate room while the clinician speaks with the caregiver. In telemedicine, however, asking a patient to step away for a private discussion is more complicated, requiring a thoughtful approach to maintain trust and privacy. As also described in the literature and noted by our team, it is essential to ensure that the patient can safely remain alone outside the room [16]. If this is not possible, a healthcare professional or another family member should be present. Most focus group participants obtained history separately from the patient and the caregiver during the same telemedicine visit, while some did so concurrently or in a separate session, though the latter added complexity. The literature and our experience agree on the importance of careful observation of patient behavior during telemedicine visits, including facial expressions, body language, and interaction with caregivers [5,13,16]. Based on the DEM-NTN experience, a structured form/template of the medical history and neurological examination elements that need to be covered during the remote assessment should be readily available.

#### 3.3.5. Neurological Examination

Regarding neurological examination, some participants referred to the necessity of oculomotor examination, speech (dysarthria, dysphonia), and gait assessment. Rigidity, muscle strength, sensory function, olfaction, deep tendon reflexes, and hearing cannot be examined in the absence of an LTHP, which aligns with the existing literature. Concerning mobility scales, all the participants mentioned the remote feasibility of unified Parkinson’s disease rating scale Part III (UPDRS III) apart from the rigidity and postural instability parts, with almost half of them (40%) mentioning that the Timed Up and Go (TUG) test is a useful additional tool. These statements agree with the DEM-NTN experience and the literature evidence, highlighting the aforementioned limitations of remote neurological examination [12,13,16]. Table 3 demonstrates the essential components of neurological examination proposed in our final protocol based on the presence or absence of an LTHP. Depending on the clinical scenario, the remote specialist can also assess Barre and Mingazzini signs, coordination, cranial nerves VII and XII, while an LTHP can additionally assess and/or perform a Romberg test, postural reflexes (pull test), muscle strength examination, cranial nerves I, II, and VIII, deep tendon and superficial reflexes, including the plantar response, frontal release signs, and sensory examination, including cortical sensory functions (stereognosis, graphesthesia, two-point discrimination), especially in suspected corticobasal syndrome, when needed.

#### 3.3.6. Neuropsychological Assessment

Most focus group participants used the MMSE as a remote screening tool, with the ACE-R and Montreal Cognitive Assessment (MoCA) also being mentioned. As the MoCA has been validated remotely in several patient populations such as Parkinson’s disease (PD), it was selected as the screening tool included in the final version of the protocol [5,17]. The Hopkins Verbal Learning Test—Revised (HVLT-R), Oral Trail Making Test (O-TMT), apraxia assessment, Boston Naming Testing (BNT), timed verbal fluency tests (semantic and phonemic), Digit Span (Forward and Backward), and the Clock Drawing Test (CDT) were reported as commonly administered, which agrees with the literature and the DEM-NTN experience [5,16]. According to a critical systematic review of tele-neuropsychological validity studies during the pandemic, cognitive screening tests, such as MMSE and MoCA, language tests, such as BNT and letter fluency, attention/working memory tasks, such as Digit Span Total, as well as memory tests, such as HVLT-R, demonstrate strong reliability and validity properties in remote settings [18]. These tools are also recommended in the final protocol in case a detailed neuropsychological assessment is needed.

While several questionnaires can be delivered remotely, focus group participants emphasized the importance of selecting neuropsychological and neuropsychiatric tools that are not only validated for telemedicine, but also efficient and minimally fatiguing for patients—an especially critical factor in remote assessments. They also noted that within the Greek healthcare system, clinician-led video instructions are generally preferred over digitized, web-based neuropsychological tools. One participant stated that “digital tools usually require out-of-pocket payment from users, and they are not financially accessible to the majority of the population.” Digital illiteracy issues were also highlighted. In this context, another participant mentioned that “many elderly patients in Greece are not familiar with technology, and the use of these tools becomes more complicated.” However, it was also highlighted that “limited familiarity with technology should not exclude the use of digital applications, as appropriate training can mitigate this barrier.” Collectively, a decision was made to include only the least complicated, video-led scales and tools in the final protocol.

#### 3.3.7. Neuropsychiatric Assessment

Most participants responded that they administer the Neuropsychiatric Inventory (NPI) scale to the caregiver, with some of them also use the Geriatric Depression Scale 15-item short form (GDS-15), which agrees with our experience and the literature [16]. The Mild Behavioral Impairment Checklist (MBI-C) was also mentioned as a tool that can be administered online, but our team has no relevant experience. As GDS-15 refers to patients over 65 years of age [19], in our protocol, the Patient Health Questionnaire-9 (PHQ-9) in younger cases is recommended [20]. Importantly, it was emphasized in focus groups that the neuropsychiatric assessment tools should be tailored to each patient’s symptoms and differential diagnostic needs to ensure a targeted and efficient evaluation within the time restrictions of the telemedicine session. Depending on the case, the Short Anxiety Screening Test (SAST) and other more specific instruments, such as the Revised Self-Monitoring Scale (RSMS), can also be administered. Finally, the incorporation of easy-to-administer questionnaires assessing health-related quality of life is recommended, such as the EuroQol-5 Dimension (EQ-5D).

#### 3.3.8. Functional Assessment

Most focus group participants mentioned that they do use functional assessment scales, such as the Lawton IADL, which is also used in the DEM-NTN and can be administered online [5].

#### 3.3.9. Dementia Staging

As identified in the literature and our experience, the Clinical Dementia Rating scale (CDR) can be administered remotely, while in dementia cases of the FTD spectrum, the CDR-FTLD can be performed [21].

#### 3.3.10. Evaluation of Laboratory and Neuroimaging Findings and Referrals for Diagnostic Work-Up

Referrals for neuroimaging and laboratory tests, lumbar puncture, and genetic testing should be completed according to the current clinical guidelines for in-person evaluation. Depending on availability, if needed, a lumbar puncture could be performed locally, and cerebrospinal fluid (CSF) samples could be sent for biomarkers (total tau, phospho-tau, amyloid-beta), and blood samples for phospho-tau217 or other testing in an appropriate laboratory. In cases of early-onset cognitive decline or familial forms, genetic testing can also be performed with appropriate genetic counseling. The results of laboratory and neuroimaging tests can be sent to the physician via email through a secure connection. When needed, the patient can also be admitted to a tertiary hospital for further investigation. The abovementioned procedures have already been successfully implemented in the DEM-NTN.

#### 3.3.11. Ethical and Legal Considerations

All three sources highlighted the importance of obtaining informed consent from patients (or their legal representatives) prior to the telemedicine visit, ensuring that they understand the nature of the assessment, benefits, limitations, potentially requiring in-person assessment for an accurate diagnosis, and their right to withdraw at any time [5,13,16]. Consent should also be obtained for transmitting medical data to the referral physician or for recording the video-teleconferencing session. The protection of patient data during transmission and storage was emphasized, as well as the need to comply with relevant data privacy regulations (e.g., General Data Protection Regulation, GDPR), including encrypted access with personal passwords to the electronic database of the medical data. Some participants mentioned obtaining verbal instead of written consent. However, based on the literature and our experience, written consent was finally included as a requirement in the protocol [5,13,16]. Privacy in the room was also mentioned as important by all three sources.

The legislative framework for telemedicine in the public sector in Greece was published in 2011 and remains unchanged to date. This describes telemedicine as a consultation tool for the local physician and not as a direct, legally established assessment of the patient. Thus, it is advised to send a prescription recommendation for medication to the patient’s local physician rather than the telemedicine physician prescribing it themselves. Similarly, issuing a disability certification for a patient who has been examined solely via telemedicine is not currently recommended.

Regarding the management of patients in relation to legal and ethical issues (such as decision-making capacity, management of financial matters, participation in daily activities such as cooking, driving, etc.), up-to-date guidelines should be followed. For the assessment of the legal capacity, when needed, it is recommended to conduct the Testamentary Capacity Assessment Tool (TCAT) [22].

#### 3.3.12. Clinician–Patient Communication

As highlighted in the focus groups, sociocultural factors must be considered, as digital illiteracy is common among older adults in remote regions of Greece. This necessitates a greater effort in communication and empathy to ensure patient and caregiver engagement and adherence to the process. Literature-based evidence and our experience also emphasized that maintaining eye contact by frequently looking at the camera instead of the screen, speaking clearly, and active listening with gestures are key factors ensuring optimal communication [5,13,16]. It is also suggested by the literature for healthcare professionals to maintain good, professional posture and allow for a 1–2 s pause to avoid overlapping speech due to slight time delays [5,13,16].

#### 3.3.13. Interconnection with Other Services

Literature evidence, focus group participants, and our team agreed that collaboration between local and remote healthcare providers is essential for optimal and continuous care [23]. The participants also emphasized the importance of healthcare providers being familiar with local services and having a clear referral plan for interconnected healthcare resources, such as tele-psychoeducation, caregiver support groups, tele-exercise programs, and social services. Additional non-pharmacological interventions mentioned by the participants included remote mindfulness programs and problem-solving interventions. One participant highlighted that the “participants can significantly benefit from cognitive and physical rehabilitation programs that are provided either remotely or in a local healthcare facility close to the patient.”

#### 3.3.14. Instructions

Focus group participants highlighted various methods for providing instructions after a telemedicine visit, with ca. one third (26.7%) preferring to schedule a follow-up appointment with the caregiver or orally deliver instructions to the patient or the caregiver. Fewer participants (13.3%) favored communication through a collaborating physician, medical center, or email. Based on our experience, a written report summarizing the findings of the assessment, diagnostic approach, and instructions for both pharmacological and non-pharmacological interventions, including regular physical exercise, participation in social activities, a Mediterranean diet, and management of cardiovascular risk factors [24], is very helpful to be sent to the referring physician. Safety measures at home, advice on driving and family planning should also be provided remotely in accordance with the current guidelines for cognitive disorders. It is also recommended to send online informational materials to caregivers on dementia and patient management. Finally, as identified by our team, clear instructions for the re-evaluation and follow-up of the patient via telemedicine should be provided to ensure regular monitoring.

#### 3.3.15. Satisfaction of the Patients, Caregivers, and Healthcare Professionals

As stated by most focus group participants, the literature, and our team, the evaluation of users’ satisfaction is of paramount importance for improving a telemedicine service [25]. Satisfaction questionnaires are suggested to be provided and anonymously completed by patients, including those with mild stages of dementia, caregivers, and healthcare professionals attending the telemedicine assessment. The questionnaire should include items regarding convenience, quality of communication, communication with the clinician, cost, and overall satisfaction with the service. In the DEM-NTN, a satisfaction questionnaire tailored to the specialized remote care for cognitive disorders was developed, which can be used with appropriate adaptations in other similar services [14].

#### 3.3.16. Training and Certification of Telemedicine Users

According to the literature, it is essential for the remote and local healthcare professionals to be familiar with the telemedicine system [26]. A training session should be conducted initially, and periodically thereafter. Based on our experience, specialized training in neurological and neuropsychological assessment in telemedicine settings would also be very helpful, and the predefined assessment protocols should be easily available, with the clinical staff also being regularly trained on them.

#### 3.3.17. Alignment with the WHO’s Seven Dimensions of Healthcare Quality

During our team’s consensus meeting, it was ensured that all the elements of the final protocol aligned with the WHO’s seven dimensions of healthcare quality: effectiveness, safety, people-centeredness, timeliness, equitability, efficiency, and integrated care. In terms of effectiveness, the remote assessment consisted of scientifically validated, evidence-based procedures and scales, and it was also stated that the new protocol would be subject to modifications and regular updates based on new scientific evidence. The protocol is patient- and caregiver-centered, as the entire process respects their values, preferences, and needs as a priority. The alternative of an in-person visit should be clearly given, the time of the assessment should be flexible, and feedback should be gathered from the users about their telemedicine experience, resulting in appropriate adjustments. Concerning timeliness, the assessment of patients via telemedicine should be conducted as soon as possible to prevent diagnostic or treatment delays, and regular follow-up with optimization of waiting times should be prioritized. For efficiency, workload should be minimized, and every effort should be made to reduce healthcare costs (for patients, caregivers, family members, and the national healthcare system) while maximizing the utilization of the existing resources (infrastructure, healthcare professionals, administrative and technological support staff). Regarding equity, patients should be assessed regardless of their demographic and cultural characteristics (age, gender, family, economic and social status, political beliefs, level of education, familiarity with technology, ownership of teleconferencing devices, Internet access, profession, religion, nationality, etc.), as well as other factors such as mobility, hearing, and vision impairments. Concerning safety, privacy, data security, and appropriate measures for ensuring patients’ safety during the assessment (e.g., the requirement of a local healthcare professional for minimizing fall risk during gait evaluation) are also highlighted in the protocol, along with a management plan for technical difficulties or communication failures. Finally, the protocol aligns with integrated care, since it emphasizes interconnection with appropriate services (tele-psychoeducation, etc.).

### 3.4. Short Form of the Final Protocol

A shortened form of the final protocol including the key components is provided in Table 4.

### 3.5. Pilot Implementation of the Protocol in the “Specialized Outpatient Clinic for Memory, Dementia, and Parkinson’s Disease Through the National Telemedicine Network”

Although the protocol was feasible and acceptable, technical challenges were encountered in 17% (2/12) of the visits during the pilot implementation period due to unstable Internet connection on certain islands (e.g., Leros), and the videoconferencing was successfully switched to a telephone call in one case. Notably, in most cases (83%), the session duration slightly exceeded the predefined 60 min. For this reason, the following adaptations were made: (i) the local healthcare professionals were asked to provide patients and family members with the detailed instructions for their participation in the tele-psychoeducation programs, as well as for the completion of satisfaction questionnaires, and logistical clarifications for diagnostic work-up when needed, (ii) the EQ-5D questionnaire was incorporated in the medical history process, (iii) an online version of the MBI-C was developed, which was distributed to local healthcare professionals and completed after the telemedicine session, either at the health center or at home, depending on the family members’ familiarity with technology, and (iv) due to the low completion rate of satisfaction questionnaires, a reminder was set for the users to complete them at the end of each telemedicine visit. Local healthcare providers appreciated the written instructions sent by our clinical team. Positive feedback was received from family members about the possibility of tele-psychoeducation sessions via our connection with the Athens Alzheimer’s Association. Overall, this pilot phase provided insights into the implementation of the protocol in real-world settings, leading to minor adjustments prior to its broader application.

## 4. Discussion

In this study, following an innovative methodological approach based on the combination of literature review, real-world experience, and focus groups among experts, we developed and piloted a comprehensive, specialized protocol for the neurological, neuropsychological, and neuropsychiatric assessment of patients with cognitive disorders through telemedicine, aligned with the seven (7) dimensions of healthcare quality as defined by the WHO. This protocol can be implemented in the context of the Greek healthcare system, and probably in other frameworks and specialties with appropriate adaptations.

This study aligns with the growing emphasis on addressing disparities in healthcare, promoting equity, inclusion, and accessibility to specialized services [27]. Telemedicine holds tremendous potential for bridging geographical barriers and reaching underserved populations [28]. By developing this protocol, our aim was to enhance equal access to evidence-based, high-quality healthcare regardless of the area of residence.

In our final protocol, we did not incorporate web-based or other digital applications for neurological or neuropsychological assessment that could enhance remote evaluations. Our approach diverges from some validation studies showing promising results for the remote use of digitized neuropsychological testing batteries, such as Mindmore Remote [29] and Brain on Track [30], as well as such tools as smartphone-based finger tapping applications for quantitative assessment of bradykinesia in Parkinsonian syndromes [31]. Our goal was to create a widely applicable protocol that does not rely on advanced or sophisticated digital tools, ensuring accessibility, minimal training for the local healthcare professionals, and ease of implementation. Our focus groups also identified that clinician-led video instructions are preferred in the context of the Greek healthcare system and Greece’s aging population with often limited digital literacy. Hence, for the suggested protocol, widely used, validated neuropsychological tools that could be administered remotely with appropriate adaptations by specialized clinical neuropsychologists were selected. Future studies could focus on including digitized tools, especially for the parts of the examination that are the hardest to assess in remote settings, such as muscle tone, strength, reflexes, sensation, as well as visuospatial and executive functions.

Patients living with cognitive disorders and their families often face significant practical and emotional challenges in their everyday lives [32]. In addition to the core assessment and diagnostic elements, our protocol emphasizes the integration of tele-psychoeducation for both patients and caregivers, aiming to improve their quality of life and coping skills. This can be achieved via connecting with institutions organizing videoconferencing sessions, online support groups, or webinars to provide information about the nature of cognitive decline, strategies for managing symptoms, and useful resources.

Given the importance of regular follow-up in cognitive disorders to monitor disease progression and adjust treatment plans [33], our telemedicine protocol highlights the importance of ensuring ongoing patient evaluation and continuous care by providing clear instructions for the follow-up visits in collaboration with primary care physicians and the administrative team of the NTN.

In addition to its clinical utility, the TeleCogNition protocol holds significant potential for generating system-level benefits, particularly in terms of cost savings, travel burden reduction, and timely diagnosis. In Greece, patients living on remote islands or in mountainous regions frequently face long distances, limited transportation infrastructure, and logistical challenges when accessing specialized memory clinics, often requiring ferry travel, overnight stays, or absence from work and caregiving duties. By delivering remote cognitive assessments directly to local health units, the protocol mitigates these barriers and minimizes the indirect costs associated with long-distance travel. The easier access to specialists enables the earlier detection of cognitive decline, facilitating timely diagnosis, appropriate intervention, improved patient outcomes and care planning. The expected cost-effectiveness lies in reducing unnecessary referrals, optimizing clinical workflows, and potentially delaying institutionalization through earlier support.

The existing evidence from Greek initiatives further supports the potential of telemedicine to deliver cost savings, reduced travel burden, and earlier diagnosis. For example, Politis et al. reported on the INTRINSIC program, an integrated mental health teleplatform deployed in low-resource primary care settings, demonstrating improved accessibility, early case detection, as well as reduction in the distance travelled and time spent on visiting mental and cognitive health services among older adults in underserved regions of Greece [15].

Our study has certain limitations. The pilot implementation, as expected, involved a relatively small sample size, which may constrain the generalizability of the initial findings. Nevertheless, this early phase was intended primarily to assess feasibility and identify practical and procedural issues that could inform future refinements. Further, while the protocol targets underserved and remote populations, variability in Internet connectivity and digital literacy, particularly in rural regions, may affect the consistency and quality of remote assessments. Although the protocol includes preparatory steps, structured technical guidance, and clinician support to minimize these barriers, such challenges remain important systemic concerns. Addressing them will require broader health policy engagement and investment in digital infrastructure to ensure equitable delivery of tele-neurocognitive services across diverse settings.

The context-specific design, tailored to the healthcare system in Greece and leveraging the NTN network, potentially limits the generalizability of the protocol to other settings. The Greek National Health System (ESY) is a mixed tax-funded national and social insurance system, including public hospitals and health centers in urban and rural areas [34]. ESY faces significant challenges including staff shortages, geographical disparities in access to specialized care, and long waiting times in public hospitals [34]. Our telemedicine protocol prioritizes accessibility and equity by leveraging the existing infrastructure of the NTN, provided to patients in the underserved Aegean islands without out-of-pocket costs. While the specific implementation details may vary, appropriate organizational and logistical adaptations can be applied to fit the local context of the healthcare system while maintaining the core principles of the protocol, such as patient-centered care, evidence-based assessment, and interdisciplinary collaboration. For instance, in market-based systems, private specialized memory centers could adopt the protocol and possibly integrate it into insurance plans. In centralized healthcare systems, the protocol could be integrated into national clinical guidelines, ensuring consistency across different regions, with standardized documentation into the existing electronic health record systems. In low-resource settings, the use of mobile technology such as smartphones and tablets instead of advanced desktop equipment, along with the creation of a step-by-step guide, could improve feasibility.

Importantly, the modular structure of the protocol, comprising distinct yet integrable neurological, neuropsychological, and neuropsychiatric components, facilitates customization to local clinical guidelines, patient populations, and resource availability. For instance, the protocol is well-suited for regions facing similar barriers to specialist care, such as rural and insular areas in Southern Europe, Mediterranean communities, and remote populations in countries such as Canada or Australia. In such contexts, where geographic isolation, limited infrastructure, and healthcare workforce shortages often delay cognitive assessment, this model offers a scalable and structured solution that promotes early detection, continuity of care, and equitable access.

Moreover, as there are no validation data at this stage, the clinical effectiveness of the protocol should be evaluated in real-world practice. The pilot implementation phase of the protocol in the DEM-NTN gave us initial critical insights into its feasibility and led to improvements. The protocol continues to be implemented in the DEM-NTN, and it is also going to be evaluated as a part of the TeleCogNition project in the following months.

Furthermore, since cost-effectiveness modeling was beyond the scope of this protocol development study, future research should also focus on specific indicators such as time saved in travel, resource utilization (staff time), and infrastructure requirements compared to standard in-person visits. Such analyses are critical to informing decisions about scalability and long-term integration into public health structures.

One key strength of this study is the comprehensive and multi-faceted methodological approach. Unlike previous efforts that may have relied solely on literature reviews or expert opinion, a combination of three critical sources of information was used: a rigorous literature review, in-depth focus groups with experienced clinicians, and direct experience from a specialized telemedicine outpatient clinic. The literature review provided a strong foundation of evidence-based practices and the existing recommendations. Our hands-on experience with a telemedicine outpatient clinic provided valuable insights serving as a real-world testing ground for the theoretical concepts identified in the literature. In addition, the participation of healthcare professionals from several geographical regions in the focus groups enhanced the feasibility and sustainability of our protocol in the Greek healthcare context. This triangulated approach, encompassing both theoretical knowledge and real-world insights, allowed us to develop a protocol that is not only evidence-based but also feasible within the healthcare system of Greece and other countries with similar structures. This iterative process involved continuous feedback loops, with the pilot implementation phase ensuring that the final protocol was based on the available evidence, tailored to the needs of patients, caregivers, and healthcare professionals in real-world settings.

Another critical strength of our protocol is the alignment with the WHO’s seven dimensions of healthcare quality, considering not only the technical and clinical requirements, but also important issues such as patient-centeredness, equity, integration, and safety. For example, great emphasis was placed on effective interconnection with other available services, such as tele-psychoeducation programs for caregivers, enabling a holistic, integrated approach.

In addition, the inclusion of user satisfaction–experience metrics allows for a more comprehensive understanding of the feasibility and acceptability of the TeleCogNition protocol in real-world settings. Assessing patients’ and clinicians’ perceptions of usefulness and communication quality provides insights into the likelihood of long-term adoption and adherence, especially in remote or underserved areas where trust in technology may vary. These metrics also help identify practical barriers, such as technical difficulties or discomfort with remote interaction, which may not be captured by clinical outcome measures alone.

In contrast to the existing Greek telemedicine initiatives such as the NTN and INTRINSIC, the TeleCogNition protocol offers a structured and standardized approach that was specifically designed to address the absence of clear official national or international guidelines for the remote assessment of cognitive disorders. While prior efforts, such as the DEM-NTN or services provided by the Athens Alzheimer’s Association, have significantly improved access to care, particularly in underserved regions, they have not used a unified clinical framework.

TeleCogNition extends these earlier efforts by introducing an integrated multidisciplinary protocol that encompasses neurological, neuropsychological, and neuropsychiatric dimensions, specifically tailored for delivery via telemedicine. Importantly, the model also incorporates critical organizational, ethical–legal, and technological considerations, as well as communication strategies between clinicians and patients, in alignment with the WHO’s framework for quality care. The protocol’s development followed a rigorous multi-phase process involving literature synthesis, field experience from the established telemedicine practice, and structured feedback from expert focus groups, ensuring both scientific grounding and real-world relevance. To our knowledge, this represents the first initiative in Greece to systematically pilot and evaluate such a comprehensive tele-assessment model for cognitive disorders.

Importantly, this standardized telemedicine protocol can serve as a valuable resource not only for healthcare professionals, but also for policymakers and researchers, by con-tributing to the development of national guidelines for the use of telemedicine in cognitive disorders. The integration of this protocol into the existing healthcare system and the NTN in Greece enhances the sustainability and scalability of this type of telemedicine initiatives, given the expected expansion of the NTN nationwide.

In Greece, numerous geographically isolated areas, including the Aegean and Ionian islands, mountainous mainland villages, and borderland territories, continue to face systemic limitations in accessing specialized cognitive care. In this context, a major strength of this protocol lies in its inherent flexibility and potential for adaptation to other underserved and remote populations beyond the pilot setting in our country.

Beyond geography, the TeleCogNition framework can be expanded to encompass a broader range of neurodegenerative and neuropsychiatric conditions. For example, individuals with PD often present with cognitive and neuropsychiatric symptoms that are under-recognized in routine care. By adapting the neuropsychological and behavioral modules, the protocol could support the remote assessment of PD-related cognitive decline, apathy, hallucinations, or impulse control disorders. Similarly, the protocol could be used to systematically detect and monitor MCI and MBI, which are often missed in standard primary care settings. This approach would allow for large-scale early detection, staging, and personalized prevention and intervention strategies.

This approach is also aligned with the WHO’s Global Action Plan on the Public Health Response to Dementia 2017–2025, which has been extended through 2030. One of the plan’s core strategic objectives is to promote early diagnosis, risk reduction, and timely access to care, particularly in underserved populations [35]. By supporting structured, scalable, and remote assessment of early cognitive and behavioral symptoms, the TeleCogNition protocol contributes directly to these global goals. Its potential to detect and monitor conditions such as MCI and MBI at a population level can enhance public health planning, facilitate earlier interventions, and help delay progression to dementia, all of which are critical targets in the international response to the dementia epidemic.

## 5. Conclusions

The present comprehensive, specialized telemedicine protocol for the assessment of patients with cognitive disorders can serve as a model that can be adapted not only to other medical specialties in Greece, but also to other healthcare systems worldwide, offering a scalable solution for delivering high-quality care through telemedicine. In this way, regardless of their area of residency, patients can have equal and timely access to specialized healthcare. Future research should focus on validating the protocol’s long-term effectiveness and exploring its integration into broader healthcare frameworks.

## Figures and Tables

**Table 1:** Sub-areas of the telemedicine visit and the questions developed for the semi-structured interview guide for the focus groups.

Telemedicine Visit Sub-Area	Questions for the Semi-Structured Interview Guide for Focus Groups
Organizational and technical aspects	1. How do you think the teleconference with the patient should begin?
	2. In your opinion, what should be the essential requirements in the patient’s examination room?
Neurological assessment (neurological examination, neuropsychiatric and functional assessment)	3. Which parts of the neurological examination do you consider essential and which could be omitted when assessing a patient with cognitive complaints via telemedicine, and why?
	4. Do you use mobility assessment scales (e.g., UPDRS III, Timed Up and Go test), and which do you consider most suitable for telemedicine evaluation?
	5. What is your experience and opinion regarding the use of neuropsychiatric symptom scales for patients with cognitive disorders through telemedicine?
	6. What is your experience and opinion regarding the use of functionality scales for patients with cognitive disorders through telemedicine?
Neuropsychological assessment	7. Which neuropsychological tests do you consider most suitable for a comprehensive neuropsychological assessment protocol via telemedicine?
	8. What is your experience and opinion regarding the use of new technologies, such as specific software, during neuropsychological assessment via telemedicine?
Ethical and legal aspects	9. Do you take a separate history from the caregiver without the patient’s presence during telemedicine assessment for patients with cognitive disorders, and how do you think this should be completed?
	10. What ethical and legal issues do you believe arise when examining patients with cognitive disorders via telemedicine, and how do you address them?
Instructions and linkage with other services	11. How would you provide instructions at the end of the telemedicine visit? Via email, and, if yes, to whom, verbally to the caregiver, or verbally to the healthcare professional present, if any?
	12. Which non-pharmacological interventions would you recommend for patients with cognitive disorders whom you assess via telemedicine (e.g., psychoeducation, caregiver support, physical and cognitive training, social services)? Do you think there should be a specific plan for linking with other entities, and if so, with whom?

Note: UPDRS III, Unified Parkinson’s Disease Rating Scale Part III.

**Table 2:** Responses to the preliminary questionnaires from the focus group participants.

Question	Physicians	Psychologists/ Neuropsychologists
1. Do you believe that developing a specialized assessment telemedicine protocol for cognitive disorders would be useful?		
Yes	100%	100%
No	0%	0%
2. In your daily practice, do you obtain the patient’s and/or caregiver’s consent before the remote assessment?		
Yes, verbal consent	75%	14.3%
Yes, written consent	25%	85.7%
No		
3. Before the remote assessment, do you require the presence of a caregiver during the session?		
Yes	100%	28.6%
Depending on the situation	0%	42.9%
No	0%	28.6%
4. Do you conduct a separate interview with the caregiver (without the patient being present) through telemedicine?		
Yes, systematically	25%	26.6%
Depending on the situation	75%	42.9%
No	0%	28.6%
5. In patients with cognitive impairment/dementia, do you systematically assess their mobility through telemedicine?		
Yes, systematically	50%	28.6%
Sometimes	50%	28.6%
Never		42.9%
6. For patients with suspected Parkinsonism or movement disorders, do you use the UPDRS III scale (excluding muscle tone and postural reflex testing) via telemedicine?		
Yes, systematically	25%	14.3%
Sometimes	25%	42.9%
Never	50%	42.9%
7. Do you systematically use neuropsychiatric assessment scales (e.g., NPI, FBI) in the telemedicine visit?		
Yes, systematically	50%	57.1%
Sometimes	50%	42.9%
Never	0%	0%
8. Do you systematically use self-assessment or informant-based questionnaires for cognitive impairment/dementia (e.g., IQCODE) via telemedicine?		
Yes, systematically	25%	0%
Sometimes	50%	57.1%
Never	25%	42.9%
9. Do you systematically use functional assessment scales (e.g., FAQ, Lawton IADL) via telemedicine?		
Yes, systematically	75%	85.7%
Sometimes	0%	14.3%
Never	25%	0%
10. Which neuropsychological tool do you use as a screening test via telemedicine?		
MMSE	50%	57.1%
ACE–R	25%	14.3%
MoCA	25%	28.6%
11. Do you personally prescribe medications to patients that you assess via telemedicine?		
Yes	100%	0%
No	0%	100%
12. Do you provide medical certificates (for example, for disability or other purposes)?		
Yes	75%	0%
No	25%	100%
13. Do you have a structured referral plan to other services (e.g., cognitive training, psychoeducation/support for caregivers, social services, etc.) for patients you assess through telemedicine and caregivers?		
Yes	75%	85.7%
No	25%	14.3%
14. Do you provide a satisfaction questionnaire to patients and caregivers after a telemedicine consultation?		
Yes	100%	14.3%
No	0%	85.7%
15. For patients with cognitive impairment/dementia whom you have assessed via telemedicine, what percentage do you believe might have received a different diagnosis if assessed in person?		
<20%	75%	85.7%
20–40%	0%	14.3%
40–60%	0%	0%
>60%	25%	0%

UPDRS III, Unified Parkinson’s Disease Rating Scale Part III; NPI, Neuropsychiatric Inventory; FBI, Frontal Behavioral Inventory; IQCODE, Informant Questionnaire on Cognitive Decline in the Elderly; FAQ, Functional Activities Questionnaire; IADL, Instrumental Activities of Daily Living Scale; MMSE, Mini–mental state examination; ACE-R, Addenbrooke’s Cognitive Examination—Revised; MoCA, Montreal Cognitive Assessment.

**Table 3:** Essential components of the neurological examination proposed in the final telemedicine protocol, based on the presence or absence of a local trained healthcare professional.

Element of Neurological Examination	Required in All Cases	Required in All Cases If a Local Trained Healthcare Professional Is Present
Posture and gait assessment	- Ask the patient to walk freely, ideally at least five steps from one side of the room to the other in front of the camera. - Ensure the entire body is captured within the camera’s field of view. - Proper lighting, stable connectivity, and a high-resolution camera and a screen are required to assess arm swing, mild tremor, or dystonia while walking. - Ask the patient to use his/her walking aids during the examination.	- Tandem walking: an LTHP close to the patient is required for safety reasons
Oculomotor examination (cranial nerves III, IV, VI)	- Smooth pursuit: Ask the patient to follow your finger on the screen in the four cardinal directions. - Saccadic eye movements: Ask the patient to look to the right, left, up (at the ceiling), and down (at the floor).	- Ask the LTHP to examine smooth pursuit
Assessment of dysarthria (cranial nerves IX, X)	As in-person	
Assessment of bradykinesia	- The patient should be seated in a chair in front of the camera. Ensure the camera captures the lower half of the body, and reposition appropriately if needed. - Finger tapping, fist opening/closing, and pronation/supination of the upper limbs: as in-person - Foot and toe tapping for the lower limbs: Ensure the lower limbs are visible	
Tremor examination	- Ensure proper positioning for the visibility of upper or lower limbs - Resting tremor: as in-person, including distraction techniques (e.g., asking the patient to count backward from 100 by sevens or ones). - Postural tremor: as in-person - Action tremor: as in-person	
Muscle tone examination	-	- Performed by an LTHP in front of the camera
Mobility scales	- In suspected parkinsonism: perform UPDRS-III, excluding rigidity and postural impairment components (items 22 and 30) - Video quality and connection speed may affect the evaluation of bradykinesia and tremor, especially in mild cases. - The patient should be repositioned in the room to ensure upper and lower limb movements are clearly visible.	- Ask the LTHP to assess muscle tone and postural reflexes

Note: LTHP, local trained healthcare professional; UPDRS III, Unified Parkinson’s Disease Rating Scale Part III.

**Table 4:** A shortened form of the final protocol.

Sub-Area of the Telemedicine Visit	Key Components
Organizational aspects	Adequate space for gait assessment Well-lit private, quiet room Available pen and A4 piece of paper Prefilled sent form with medical history and medications No calendars and mobile phones close to the patient
Technical aspects	High-definition camera and screen, noise-canceling microphone, safe and stable Internet connection, screensharing, direct access to technical support, alternative communication mode in case of connectivity disruption, documentation in encrypted electronic database that is shared between local and remote healthcare professionals
Initiation of the telemedicine session	Greet the participants, ask if they can hear you Introduce yourself and the other team members Verify patient’s name, date of birth Ask and record other attendees’ names Keep the patient’s address and phone number in case of an emergency Ask about any vision/hearing problems, encourage the use of eyeglasses and/or hearing aids Ensure all the participants are within the camera’s field of view
Medical history	Obtain focused, detailed medical history as in-person, first from the patient Obtain family history With the patient’s permission, obtain history from the family member/caregiver either at the same session or separately
Neurological examination	Gait assessment: ask the patient to walk freely in front of the camera (ca. 5 steps in both directions) Perform smooth pursuit (use your finger) and saccadic eye movements examination Examine dysarthria as in-person Examine bradykinesia, tremor as in-person (ensure that the body part of interest is fully captured by the camera) Perform modified UPDRS III in case of parkinsonism (excluding muscle tone and postural reflex testing) If a local trained healthcare professional is present and/or depending on the clinical scenario: tandem walking, muscle tone, postural instability, coordination evaluation, Romberg testing, assessment of the rest of cranial nerves, reflexes, frontal release signs, Barre and Mingazzini signs, sensory examination (including stereognosis, graphesthesia, two-point discrimination)
Neuropsychological assessment	MoCA as a screening tool For a detailed assessment when needed: semantic and phonemic verbal fluency, CDT, JLO, Digit Span (Forward and Backward), O-TMT (A and B), HVLT-R, BNT, apraxia testing
Neuropsychiatric assessment	NPI, GDS-15 in patients ≥ 65 years PHQ-9 in patients ≤ 65 years MBI-C in subjective cognitive decline or MCI Short Anxiety Screening Test in suspected anxiety EQ-5D
Functional assessment	Lawton IADL
Dementia staging	CDR, CDR-FTLD
Evaluation of laboratory and neuroimaging findings and referrals for diagnostic work-up	Referrals for diagnostic work-up based on the up-to-date guidelines Blood or CSF samples sent for analysis (AD biomarkers, genetic testing, other tests) Referrals for hospital admission for further evaluation when needed
Ethical and legal considerations	Written informed consent GDPR-compliant platform, encrypted access to the electronic database, data security Privacy in the room TCAT when needed
Clinician–patient communication	Look frequently at the camera Show empathy, use gestures Speak clearly and slowly
Interconnection with other services	Structured plan for referrals to cognitive rehabilitation, tele-exercise, speech therapy, caregiver support groups or social services, tele-psychoeducation
Instructions	Provision of a written medical report to the referring physician Advice on non-pharmacological interventions, safety measures at home, driving and family planning, and genetic counseling in accordance with the current guidelines Provision of online informational materials on dementia Clear instructions for the re-evaluation and follow-up
Satisfaction questionnaires	Ask the patients, family members/caregivers, and local healthcare professionals to complete satisfaction questionnaires regarding their experience of the telemedicine visit
Training of the local and remote healthcare professionals	Training of the local and remote healthcare professionals on the telemedicine system (initial and regular sessions) Regular training of the team on the telemedicine protocol
Alignment with the WHO’s seven dimensions of healthcare quality	The key elements of the protocol are aligned with the WHO’s seven dimensions of healthcare quality: effectiveness, safety, people-centeredness, timeliness, equitability, efficiency, and integrated care

Note: MBI-C, mild behavioral impairment checklist; WHO, World Health Organization; TCAT, Testamentary Capacity Assessment Tool; GDPR, General Data Protection Regulation; MCI, mild cognitive impairment; CDT, Clock Drawing Test; JLO, Judgment of Line Orientation; O-TMT, Oral Trail Making Test,; HVLT-R, Hopkins Verbal Learning Test—Revised; BNT, Boston Naming Testing; NPI, Neuropsychiatric Inventory; GDS-15, Geriatric Depression Scale 15-item short form; PHQ-9, Patient Health Questionnaire-9; CDR, Clinical Dementia Rating scale; FTLD, frontotemporal lobar degeneration; IADL, Instrumental Activities of Daily Living Scale; EQ-5D, EuroQol-5 Dimension; UPDRS III, Unified Parkinson’s Disease Rating Scale Part III.

## Data Availability

The data presented in this study are available upon request from the corresponding author due to privacy reasons.
